# Pre-Conception Androgen Levels and Obstetric Outcomes in Polycystic Ovary Syndrome: A Single-Center Retrospective Study

**DOI:** 10.3390/diagnostics14192241

**Published:** 2024-10-08

**Authors:** Mónika Orosz, Fanni Borics, Dávid Rátonyi, Beáta Vida, Szilvia Csehely, Attila Jakab, Luca Lukács, Rudolf Lampé, Tamás Deli

**Affiliations:** 1 Department of Obstetrics and Gynaecology, Faculty of Medicine, University of Debrecen, Nagyerdei krt. 98, 4032 Debrecen, Hungary; orosz.monika@med.unideb.hu (M.O.); csehely.szilvia@med.unideb.hu (S.C.);; 2 Department of Internal Medicine, Faculty of Medicine, University of Debrecen, Nagyerdei krt. 98, 4032 Debrecen, Hungary; borics.fanni@med.unideb.hu

**Keywords:** polycystic ovary syndrome, in vitro fertilization, gestational diabetes mellitus, androgen levels, pre-pregnancy hormone levels, obstetric outcomes

## Abstract

Hyperandrogenism is a determining diagnostic factor for PCOS. If pregnancy is conceived, it is considered high-risk due to several potential complications, but the correlation between pre-pregnancy androgen levels and obstetric outcomes is poorly characterized. Objective: To determine if pre-pregnancy serum androgen concentrations and androgen indexes differed when certain obstetric and neonatal outcomes appeared in PCOS. Methods: A single-center, retrospective study was carried out. All patients were treated between 2012 and 2019. A total of 73 patients had all the endocrine and obstetric data available. Pre-pregnancy hormone levels (total testosterone-T, androstenedione-AD, DHEAS (dehydroepiandrosterone sulfate), SHBG (sex-hormone-binding globulin), and TSH (thyroid-stimulating hormone) were collected, and T/SHBG, AD/SHBG, DHEAS/SHBG, T/AD indexes were calculated and compared. Results: When miscarriage was present in the history, significantly elevated pre-pregnancy AD levels were observed. Higher pre-pregnancy AD level was noted in PCOS patients delivering female newborns as compared to males. Additionally, a higher T/AD ratio was associated with subsequent preterm delivery, but significance was lost after age adjustment. Maternal age at delivery had a significant negative correlation with pre-pregnancy DHEAS levels and DHEAS/SHBG ratio. Pre-pregnancy SHBG displayed a significant negative correlation, while pre-pregnancy androgen/SHBG ratios exhibited positive correlations with both birth weight and birth weight percentile. Conclusions: Based on our data, AD and the T/AD ratio emerge as distinctive factors in certain outcomes, implying a potential specific role of altered 17-β-HSD (17β-hydroxysteroid dehydrogenase) enzyme activity, possibly influencing offspring outcomes. The pre-pregnancy T/SHBG ratio exhibits a potentially stronger correlation with fetal growth potential compared to SHBG alone. DHEAS and maternal age at delivery are strongly correlated in PCOS patients.

## 1. Introduction

Polycystic ovary syndrome (PCOS) is the most common endocrinological disease worldwide among women of childbearing age [1]. The prevalence of the disease is 8–15%; however, according to some studies, it can even reach 20% [2]. The clinical picture is characterized by the well-known polycystic appearance of the ovaries, the presence of clinical and/or biochemical features of hyperandrogenism, and the presence of oligo-anovulation and/or oligo-amenorrhea. If two of these criteria are present and other endocrinological diseases are excluded, a diagnosis of PCOS can be established according to the Rotterdam criteria [3]. In adolescence, hyperandrogenism and menstrual disorder must be present together to confirm the existence of the disease, and polycystic ovary morphology should not be used to confirm the diagnosis [2]. Polycystic ovary syndrome is a complex, multifactorial endocrine and metabolic disease that can lead to long-term health consequences. The incidence of diabetes mellitus, gestational diabetes, cardiovascular disease, obstructive sleep apnea (OSA), and endometrial carcinoma has increased [4]. Infertility affects an estimated 40–50% of women with PCOS [5]. In anovulatory infertility, PCOS may be the cause in 80% of cases [6,7]. Fertility treatments aim to restore physiological, unifollicular ovulation. In many cases, lifestyle changes are not enough, and ovulation induction may be required (letrozole, clomiphene citrate, metformin, recombinant gonadotropins) [8,9]. Multiple pregnancies are more common when using these methods. In all cases, we must strive to avoid ovarian hyperstimulation syndrome (OHSS). It is well-established that pregnancy complications occur more frequently in those who get pregnant with PCOS. Miscarriages are more common in people with PCOS, although the reason for this is not fully understood [10]. The rate of spontaneous abortions is 20–40% higher than in the average population [11]. Even at the later stages of pregnancy, there is a higher risk of developing complications, including gestational diabetes (GDM), pregnancy-induced hypertension (PIH), preeclampsia (PE), and premature birth. The rate of cesarean sections is also increased [12,13,14].

Hyperandrogenism is a determining diagnostic factor for polycystic ovary syndrome [15]. The physical symptoms of hyperandrogenemia (hirsutism, acne) may be absent, even with elevated androgen levels, and, vice versa, hyperandrogenism may well occur with apparently normal serum androgen levels. The main androgens circulating and measurable in the blood in women are dehydroepiandrosterone sulfate (DHEAS), androstenedione (AD), and testosterone (T) [16,17]. T and the locally produced dihydrotestosterone (DHT) exert their biological effects by directly binding to androgen receptors, while DHEAS, DHEA, and AD become biologically active by converting to testosterone and dihydrotestosterone [17]. The main testosterone source is the luteinized theca cells of the ovary and stromal cell hyperplasia, but, to a lesser extent, the adrenal cortex can also contribute to increased androgen production through DHEAS production [18]. AD is produced in both the ovaries and the adrenal cortex. SHBG is a protein produced in the liver; its production is significantly affected by changes in insulin as well as androgen levels [19]. A higher rate of abnormalities in early trophoblast invasion and placentation was observed in patients with hyperandrogenism and oligo-anovulation, independently of the presence of polycystic ovaries, thus suggesting a key role of hyperandrogenism in placentation [20]. In accordance with this, Sun et al. (2012) have demonstrated in a murine model that the treatment with testosterone during gestational days 16–19 reduces placental and fetal weight and increases placental steroidogenesis. These data suggest that PCOS could affect placental and fetal development and can lead to placental abnormalities and pregnancy complications [21].

Hyperinsulinemia, which is present in most cases of PCOS, increases androgen synthesis in theca cells and reduces the synthesis of hormone-binding proteins in the liver (SHBG, IGFBP-I). Due to the low SHBG level, the amount of circulating free androgens also increases, and furthermore, due to the reduced level of IGFBP-I, the IGF-1 level increases, which further increases the androgen production of the ovary [22]. Although the significance of hyperandrogenism is clear in PCOS, and adverse obstetric outcomes are also well known, the relation between the two is far from fully understood.

In this study, we aimed to find a connection between the pre-pregnancy androgen levels and obstetric and neonatal outcomes. However, total androgen levels are not the only indicators that may be used. The ratio of testosterone to androstenedione (T/AD) is the indicator of the action of 17-β-hydroxysteroid dehydrogenase (17-β-HSD), a key enzyme in AD to testosterone conversion, that has been found to be related to metabolic health in PCOS [23,24,25] and genital differentiation [26,27,28]. Also, SHBG is a key player in determining the biologically active proportion of androgens (e.g., T/SHBG = FAI, free androgen index), but the SHBG level is regulated by several factors. Furthermore, thyroid function is known to strongly affect clinical hyperandrogenic symptoms. Thus, not only detailed total androgen levels but also the T/AD ratio, the androgen/SHBG ratio, and TSH levels were examined, which is unique in the literature.

## 2. Materials and Methods

**Figure 1:**
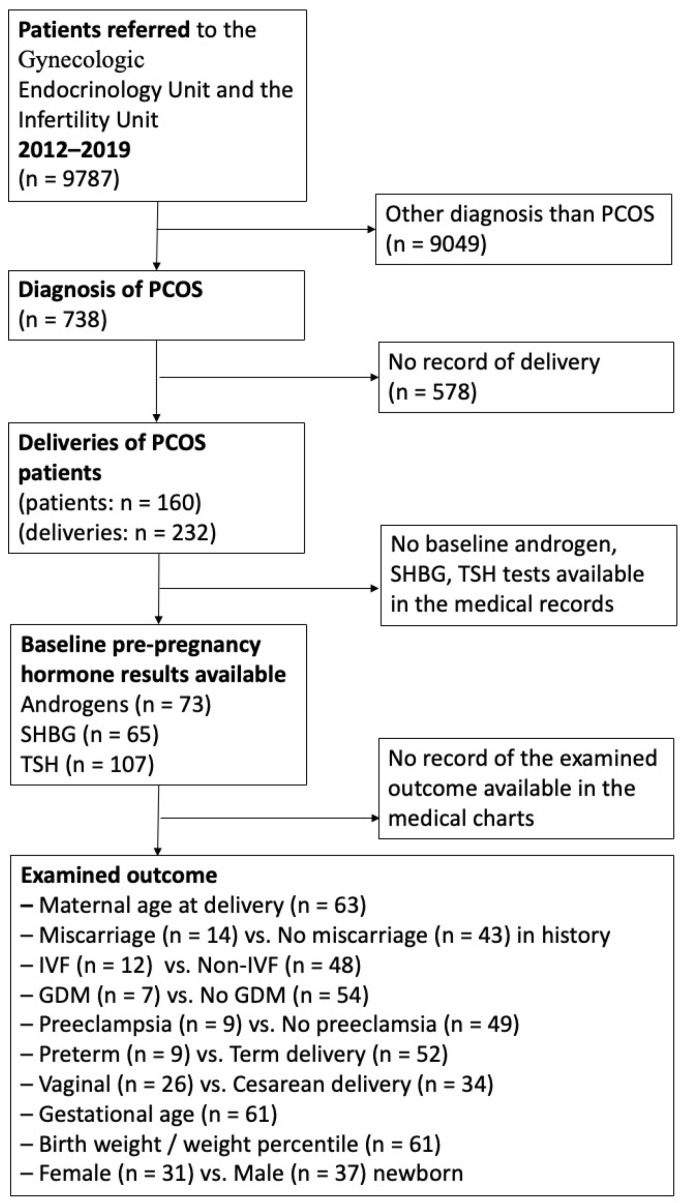
Flow chart of patient enrollment and follow-up.

Electronic medical charts of all patients treated at the Infertility Unit or the Gynecologic Endocrinology Unit of the Department of Obstetrics and Gynecology of the University of Debrecen between 2012 and 2019 were searched (Figure 1).

Inclusion criteria: the diagnosis of PCOS was established according to the Rotterdam criteria [3]. This required the presence of at least two of the following three criteria: (1) clinical and/or biochemical evidence of hyperandrogenism, (2) oligo-ovulation and/or anovulation, and (3) polycystic ovaries observed via ultrasonography. All patients (1) diagnosed with PCOS, (2) having had at least one successful delivery beyond gestational week 23, and (3) having the relevant androgen levels available at baseline before pregnancy were included. Not all hormone levels were available in the case of all patients, given that in some referred cases, they had had the laboratory examinations made elsewhere, and they were not possible to track down and were not mentioned in the documentation either or laboratory androgen tests were not carried out as no therapeutic consequence was expected from the results. However, all patients who had a given test available were included in this study. All our patients had pregnancy care and delivered at the Department of Obstetrics and Gynecology of the University of Debrecen; therefore, obstetric and neonatal data could also be collected.

Exclusion criteria included insufficient available data or the presence of any of the following: congenital adrenal hyperplasia, hyperprolactinemia, Cushing’s syndrome, androgen-secreting tumors, Hashimoto’s thyroiditis, hyperthyroidism, and hypothyroidism [3].

Clinical data were collected retrospectively through the E-MedSolution electronic medical database system and the Hungarian National Electronic Medical Database System (EESZT, Elektronikus Egészségügyi Szolgáltatási Tér). The following obstetric data were gathered from the medical history of the patients with PCOS: mode of conception, miscarriage in the medical history, maternal age, BMI, length of gestation, mode of delivery (vaginal, cesarean section), newborn gender, birth weight and birth weight percentile, and pregnancy complications (gestational diabetes, preeclampsia, preterm delivery).

Laboratory tests: Pre-pregnancy baseline total testosterone, androstenedione, DHEAS, SHBG, and TSH were collected at the time of diagnosis. T/SHBG, AD/SHBG, DHEAS/SHBG, and T/AD ratios were also calculated. Hormone levels, including total testosterone (T) (nmol/L), dehydroepiandrosterone sulfate (DHEA-S) (µmol/L), sex-hormone-binding globulin (SHBG) (nmol/L), and androstenedione (µg/L), were measured prior to pregnancy and before initiating any kind of endocrine treatment. Blood samples for hormone measurements were taken in the morning between the third and fifth days of the menstrual cycle. Anthropometric data, including age, height, and weight, were collected from pre-pregnancy records. BMI was calculated using metric units (unit of BMI = kg/m^2^).

Serum thyroid-stimulating hormone, dehydroepiandrosterone sulfate, sex-hormone-binding globulin, and testosterone were measured using an electrochemiluminescence (ECLIA) immunoassay (Roche Diagnostics GmbH, Mannheim, Germany). Serum androstenedione was measured using a chemiluminescence (CLIA) immunoassay (DiaSorin Inc., Stillwater, MN, USA).

The intra-assay and inter-assay coefficients of variation (CVs) for CLIA (Chemiluminescent Immunoassay) from DiaSorin Inc., Stillwater, MN, USA, and ECLIA (Electrochemiluminescence Immunoassay) from Roche Diagnostics GmbH, Mannheim, Germany, were both below 10%, indicating high reproducibility.

### Statistical Analysis

Statistical analysis was performed using the IBM SPSS Statistics for Windows Version 25.0 software (IBM Corp., Armonk, NY, USA). For continuous variables, the Kolmogorov–Smirnov test was used to check the normality of the distribution, and Levene’s test was used to determine the equality of variances. For parametric variables, an independent-sample *t*-test was used to compare the equality of means, and Pearson’s correlation was used to determine the degree of association. For non-parametric variables, Spearman’s correlation for association and Mann–Whitney *U* test for comparison of means were used. Nominal variables were compared using the chi-squared test or Fisher’s exact test. Logistic regression was used to determine odds ratios and age-adjusted odds ratios where significant differences were found between variables. A *p*-value less than 0.05 was considered statistically significant. Data are shown as mean ± SD or as odds ratio (OR), age-adjusted odds ratio (aOR), and 95% confidence interval, where applicable. Familywise error rate due to multiple comparisons was FWER = 1 − (1 − *p*)^7^ = 0.302 (30.2%).

## 3. Results

We screened data from 9787 patients who attended the Infertility Unit or the Gynecologic Endocrinology Unit at the Department of Obstetrics and Gynecology at the University of Debrecen between 2012 and 2019. Among them, 738 patients were diagnosed with polycystic ovary syndrome (PCOS). In our database, we focused on 232 deliveries involving 160 PCOS patients who received antenatal care at our outpatient clinic and delivered at our institute during the specified period (Figure 1).

### 3.1. Miscarriage

Pre-pregnancy androstenedione levels were significantly higher (3.60 ± 1.97 vs. 2.68 ± 1.22, *p* = 0.04) in individuals with at least one miscarriage in their medical history (Table 1). The OR for miscarriage per one unit change in AD turned out to be 1.513, 95% CI 0.988–2.317, *p* = 0.057), and after adjustment for age, it crossed the significance level (aOR 1.555, 95% CI 1.005–2.407, *p* = 0.047). However, no significant differences were found in the other pre-conception androgen levels (total testosterone, dehydroepiandrosterone sulfate), androgen/SHBG ratios, T/AD ratio, TSH levels, age, or BMI between the two groups.

### 3.2. Mode of Conception—In Vitro Fertilization (IVF)

No significant differences were noted in androgen levels, androgen/SHBG ratios, or T/AD ratio between PCOS patients who conceived without IVF (n = 48) and those who required IVF treatment (n = 12) (Table 1). However, DHEAS and androstenedione levels had a trend to be lower, and SHBG levels tended to be higher in the IVF treatment group, though these results did not reach statistical significance (AD: 2.31 ± 1.09 vs. 2.93 ± 1.41, *p* = 0.16; DHEAS: 6.45 ± 3.01 vs. 8.05 ± 3.30, *p* = 0.13; SHBG: 72.01 ± 54.40 vs. 54.73 ± 37.85, *p* = 0.20). Significantly higher age was observed in patients undergoing IVF (32.00 ± 5.26 vs. 28.83 ± 4.44, *p* = 0.027). The OR for IVF with a one-year increase in maternal age was found to be 1.157 (95% CI 1.006–1.336, *p* = 0.041).

### 3.3. Gestational Diabetes Mellitus (GDM)

No significant differences were found in pre-pregnancy androgen levels, androgen/SHBG ratios, T/AD ratio, or TSH levels between patients who developed GDM during pregnancy and those who did not (Table 1). SHBG levels tended to be lower in the GDM group, but this difference did not reach statistical significance (SHBG: 38.51 ± 22.5 vs. 61.86 ± 44.43, *p* = 0.18). The age of patients with GDM was significantly higher (32.22 ± 4.06 vs. 28.85 ± 4.70, *p* = 0.044), but no difference in BMI was observed between the two groups.

### 3.4. Preeclampsia

Pre-pregnancy androgen levels were not significantly higher in the preeclampsia group (Table 1). No significant differences were found in SHBG levels, androgen/SHBG ratios, or TSH levels between the preeclampsia and non-preeclampsia groups. However, the preeclampsia group had significantly higher BMI (32.12 ± 3.05 vs. 27.66 ± 4.58, *p* = 0.046), with no difference in age between the two groups.

### 3.5. Fetal Gender

PCOS patients delivering female newborns had a significantly higher pre-pregnancy androstenedione level (3.57 ± 1.96 vs. 2.58 ± 1.12, *p* = 0.01) (Table 2). The OR for a female newborn per one unit change in AD was 1.512 (95% CI 1.037–2.207, *p* = 0.03), and it remained significant after adjustment for age (aOR 1.495, 95% CI 1.016–2.198, *p* = 0.04). Additionally, the T/AD ratio was almost significantly lower in this group. No significant differences were identified in other pre-pregnancy androgen levels, SHBG levels, androgen/SHBG ratio, TSH levels, maternal age, or BMI between the groups delivering female or male babies.

### 3.6. Gestational Age—Fetal Maturity

No significant difference in pre-pregnancy androgen levels was observed in PCOS patients with preterm or term delivery (Table 2). However, the T/AD ratio was nearly significantly higher in those who subsequently had a preterm neonate (0.82 ± 0.61 vs. 0.55 ± 0.31, *p* = 0.052). After adjustment for maternal age, the difference was not significant, but the trend remained unchanged: OR 4.531 (95% CI 0.834–24.612, *p* = 0.08; aOR 4.173, 95% CI 0.727–23.960, *p* = 0.10). No significant differences were found in SHBG levels, androgen/SHBG ratios, and TSH levels.

### 3.7. Mode of Delivery

No significant differences were detected in pre-pregnancy androgen levels, androgen/SHBG ratios, or T/AD ratios in PCOS patients based on the mode of delivery (Table 2). Pre-pregnancy TSH levels in PCOS patients tended to be higher within the normal range in the group that underwent a subsequent cesarean section (1.77 ± 0.67 vs. 2.21 ± 1.08, *p* = 0.09), but the difference just missed statistical significance.

### 3.8. Maternal Age at Delivery

**Figure 2:**
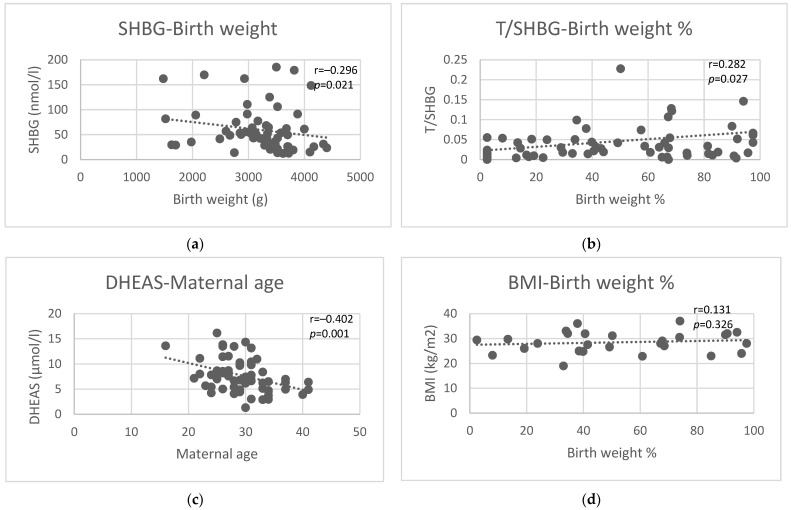
Correlations of pre-pregnancy SHBG with birth weight (**a**), pre-pregnancy T/SHBG ratio with birth weight percentile (**b**), pre-pregnancy DHEAS with maternal age at delivery (**c**), and maternal BMI with birth weight percentile (**d**).

Maternal age at delivery demonstrated a significant negative correlation with pre-pregnancy DHEAS (r = −0.40, *p* = 0.001) (Figure 2c) and DHEAS/SHBG ratio (r = −0.32, *p* = 0.01) but not with other pre-pregnancy androgen levels, androgen/SHBG ratios, T/AD ratio, or TSH levels (Table 3).

### 3.9. Gestational Age

No significant correlation was observed between gestational age at delivery and pre-pregnancy androgen levels, androgen/SHBG ratios, T/AD ratio, or TSH levels (Table 3).

### 3.10. Birth Weight and Birth Weight Percentile

Pre-conception androgen levels were not correlated with either birth weight or birth weight percentile (Table 3). Pre-pregnancy SHBG showed a negative correlation (r = −0.30, *p* = 0.02, Figure 2a), while pre-pregnancy androgen/SHBG ratios showed a positive correlation (T/SHBG (r = 0.28, *p* = 0.03), AD/SHBG (r = 0.24, *p* = 0.06), DHEAS/SHBG (r = 0.26, *p* = 0.04)) with birth weight. Pre-conceptional TSH showed no correlation with birth weight. Similar results were found for birth weight percentiles, where pre-pregnancy SHBG showed a negative correlation (r = −0.27, *p* = 0.04), and pre-pregnancy androgen/SHBG ratios showed a positive correlation (T/SHBG (r = 0.28, *p* = 0.03, Figure 2b), AD/SHBG (r = 0.25, *p* = 0.05), DHEAS/SHBG (r = 0.27, *p* = 0.03)) with birth weight percentile. In our study population, pre-pregnancy BMI was not significantly correlated with either birth weight or percentile of birth weight (Figure 2d).

## 4. Discussion

To the best of our knowledge, this is the first report comparing pre-pregnancy levels of various androgens and their ratios in groups with various obstetric and neonatal outcomes following successful pregnancies of patients with PCOS. In our database of 160 PCOS patients with 232 deliveries, we identified 73 patients whose detailed pre-pregnancy androgen levels, including total testosterone, dehydroepiandrosterone sulfate, androstenedione, sex-hormone-binding globulin, as well as TSH levels, were available. We also examined T/SHBG (=FAI), DHEAS/SHBG, and AD/SHBG since SHBG has triple significance as an indicator in PCOS: its level is decreased by androgens; insulin as a marker of the metabolic state and hyperinsulinemia common in PCOS also decreases SHBG levels; and binding of androgens (especially testosterone, see FAI) to SHBG essentially affects the biological activity of androgens. Therefore, androgen/SHBG ratios can be expected to convey further information about the clinical characteristics of the disease than androgen levels by themselves, as they incorporate different abnormalities seen in PCOS. Similarly, extra information can theoretically be expected from the T/AD ratio, too, since it can be used as an indicator of the 17-β-hydroxysteroid dehydrogenase (17-β-HSD) activity, converting AD to T. Thus, we wanted to see if these androgen-derived ratios were different in their tendencies from the individual androgen levels, and whether they could appear as useful predictive markers of the examined outcomes.

Former studies analyzing obstetric and neonatal outcomes in PCOS were more generalized in one or more aspects. Many of them analyze the following:

(1) Only the presence of PCOS as an “umbrella diagnosis” without attempting to differentiate according to the various manifestations [29];
(2) Generally, “hyperandrogenism” or “hyperandrogenaemia” [29,30] or “PCOS phenotypes” (implicitly differentiating the ones with or without hyperandrogenism), which indirectly means the analysis of the presence of clinical or laboratory hyperandrogenism [31,32,33];
(3) Androgen levels at certain points during pregnancy, but not pre-pregnancy levels [34,35,36];
(4) T/AD ratio is usually not reported [37], and even if sparsely it is, either the context of metabolic health [23,24,25] or the relation to newborn gender [26,27,28] is examined, but not other obstetric outcomes.

The findings of these studies are discussed below in comparison with the results of this study.

In this study, only pre-pregnancy androstenedione levels were significantly higher in PCOS patients who had at least one miscarriage in their history. This proved, however, independent of age and BMI as confounding factors (Table 1). The effect of androgens on endometrial receptivity is well-established, and even molecular actors (Wnt and CDK signaling pathway, HOXA gene, integrin aVβ3, MECA-79, MAGEA-11) have been identified, as reviewed in a recent paper by Yusuf et al. [38]. Early in vitro studies proved that AD decreased endometrial growth (as indicated by decreased (3)H-thymidine uptake and Ki67 expression), as well as endometrial secretory activity (glycodelin A secretion), whereas testosterone, dihydrotestosterone, and DHEA did not have this effect [39]. If miscarriage as the clinical outcome measure was examined, it was again AD that was found to differ from the other androgens in being elevated in patients with recurrent miscarriage, although not in a purely PCOS population [40]. Furthermore, in a PCOS population receiving IVF treatment with the GnRH protocol, the miscarriage rate was also found to be associated with basal AD levels as well as BMI [41]. Our results are in line with these findings: pre-conception AD was the only androgen that was elevated in the group of PCOS patients that had a miscarriage in their history. None of the other examined androgens or androgen/SHBG ratios were different in the two groups.

There was no significant difference in the baseline pre-conception androgen levels and the androgen/SHBG or testosterone/AD ratios in those PCOS patients that could conceive without IVF treatment and the ones that required IVF, although DHEAS and androstenedione levels had a trend to be lower and SHBG levels higher, even if not significantly, in those who finally had an IVF conception. The differences in pre-pregnancy androgen levels were not significant even though the IVF population has a significantly higher age, and age does correlate with DHEAS levels (see below). It is noteworthy that during IVF treatment, DHEA is widely used to improve outcomes in poor responders and those with diminished ovarian reserve, and several studies and meta-analyses support this routine [42,43], although the question is not completely settled. Nevertheless, baseline androgen concentrations have not been shown clearly to correlate with IVF success [44]. Our results suggest that probably androgen levels before conception are not useful in predicting if the treatment of PCOS-related infertility will require IVF.

We did not see a significant difference in the pre-pregnancy androgen or SHBG levels in our PCOS patients who did or did not develop gestational diabetes. This is somewhat surprising with regard to SHBG since it is well-established that low SHBG level is a good predictor of gestational diabetes [45,46,47,48]. In our gestational diabetes group, we also had this trend, but it did not reach significance. A possible explanation for this could be that the gestational diabetes group was significantly older (32.22 ± 4.06 vs. 28.85 ± 4.70 years). It is known that SHBG levels reach a nadir in late adolescence and rise with age progressively thereafter [49]. Thus, if the average age of the gestational diabetes group had been lower (if the two groups were comparable with regard to age) and, therefore, the age-specific lower level of SHBG had also been present, the difference might have reached significance, since even now the trend is obvious and nearly significant. Age matching, however, was unfortunately not possible because of the low number of cases in the gestational diabetes group.

All pre-conception androgen levels (total testosterone, androstenedione, and DHEAS) were non-significantly higher in our preeclampsia group compared to those who did not develop preeclampsia. Results in the literature correlating pre-conception androgen levels, the presence of hyperandrogenism without PCOS, and the presence of the hyperandrogenic phenotype of PCOS with the development of preeclampsia during pregnancy are contradictory. Hyperandrogenism was more common, and FAI was higher, but the individual androgen levels showed no association with preeclampsia in the study by Christ et al. [37]. Others found a correlation between preeclampsia and hyperandrogenemia [30] but not with hyperandrogenism or hyperandrogenic phenotype [33]. We also did not find a significant difference in SHBG levels of the groups that later did or did not develop preeclampsia, which is in line with the findings of other groups demonstrating no predictive value of prepregnancy SHBG levels for preeclampsia in the merged data from two large prospective cohort studies, mentioned above [37], or in the study by Spencer et al., analyzing the correlation of first trimester SHBG levels with subsequent preeclampsia [50]. However, this again is a contradictory question, as other groups found that low SHBG was a predictor of preeclampsia in infertile PCOS patients [51], and in an Iranian case–control study, SHBG was even claimed to be a predictor of preeclampsia independent of insulin resistance [52]. Our results regarding preeclampsia, however, may be confounded by the fact that the preeclampsia group had a significantly higher BMI, which is a well-known risk factor for preeclampsia as well as insulin resistance, and the latter may influence SHBG levels.

Pre-pregnancy androstenedione levels were significantly higher, and testosterone/androstenedione ratios (T/AD) were nearly significantly lower in PCOS patients who delivered a female newborn, while none of the other parameters were different between the groups delivering female or male babies. T/AD ratio is considered the marker of the activity of the 17-β-hydroxysteroid dehydrogenase (17-β-HSD) enzyme, converting AD to T. Alterations in the T/AD ratio have been examined in relation to metabolic health in PCOS [23,24,25]. Furthermore, male undermasculinization and a form of 46XY disease of sexual differentiation (DSD) caused by 17-β-HSD type 3 deficiency are associated with a decreased T/AD ratio [26,27,28]. Nevertheless, to the best of our knowledge, this study is the first where pre-conception maternal AD levels and T/AD ratios are demonstrated to be different in PCOS patients according to the gender of their offspring. One can hypothesize that an altered function of the 17-β-HSD enzyme might be present in some PCOS patients, and this modified-activity enzyme may be passed on to their fetuses, affecting their gender assignment. For example, serine phosphorylation alterations can be hypothesized, a theory used to explain some symptoms, especially hyperandrogenism and insulin resistance seen in PCOS. Most probably, it is not the altered maternal hormone levels that affect the fetal gender since the placental aromatase protects the fetus from excess androgen exposure.

Interestingly, although no difference was observed in pre-pregnancy androgen levels in those PCOS patients who had a preterm or term delivery, the T/AD ratio was higher in those who later had a premature newborn, and the trend remained present even after adjustment for maternal age. Data regarding preterm delivery and pre-pregnancy androgen levels are sparse in the literature. In the study mentioned above, Christ and colleagues found that testosterone and hyperandrogenism but not the other androgens were predictors of preterm delivery [37], whereas another study found no correlation between hyperandrogenism and preterm delivery in cases of dichorionic twin pregnancies in PCOS [53]. Neither paper reported the T/AD ratio, though. Our data suggest that even though pre-pregnancy androgen levels were comparable in the preterm and term delivery groups, the T/AD ratio and, thus, the difference in the maternal 17-β-HSD enzyme activity may have an effect on preterm birth. If, however, the gestational age was correlated to the T/AD ratio or the individual androgen levels or androgen/SHBG ratios, we did not see a significant correlation. Yet, this is not a contradiction, considering the differences between the pathophysiological background of preterm labor and the physiological factors that determine the time when term deliveries normally begin. The T/AD ratio may be related to the first but not the latter.

No pre-pregnancy difference in androgen levels or ratios could be observed according to the mode of delivery. This is in line with former studies in which androgen levels or hyperandrogenism were examined [31,33]. More general approaches of PCOS without specific examination of androgens did, however, find an increased risk of cesarean delivery [29] (elective cesarean section odds were increased significantly by 10% and emergency cesarean section risk by 7% in PCOS patients in general), but this does not contradict our and other’s findings of the missing link between altered androgen levels and cesarean delivery, because the metabolic complications in some subgroups of PCOS clearly increase the risk of gestational diabetes, preeclampsia or preterm delivery, each of which leads to an increased cesarean section risk in PCOS in general. We did not find it reported in the literature, but it is worth mentioning that, in our PCOS population, the average TSH level was almost significantly higher within the normal TSH range as compared to the vaginal delivery group, and this was probably not due to the mode of conception (IVF vs. non-IVF), maternal age, pregnancy complications, maturity or birth weight, as in these comparisons TSH levels were comparable between the respective groups.

A significant correlation was observed between the age of mothers at delivery and DHEAS and the DHEAS/SHBG ratio, but no other androgen levels among our PCOS patients. DHEAS alone proved to have both a stronger and more significant negative correlation to maternal age as compared to the DHEAS/SHBG ratio. The relation between the two variables is probably bidirectional: DHEAS levels are known to keep decreasing after young adulthood in both males and females [54], and this has been described in PCOS populations, too [55]. On the other hand, lower DHEAS levels may affect fertility negatively, leading to higher age at delivery on the one hand and also making it possible for DHEA supplementation during fertility treatment of patients with diminished ovarian reserve to exert a beneficial effect, as discussed above.

In this study, pre-pregnancy androgen levels did not correlate with birth weight. Former studies pointed to elevated testosterone levels being associated with intrauterine growth restriction, but these studies examined androgen levels at various stages of pregnancy and not before conception [56,57]. On the contrary, pre-pregnancy SHBG showed a negative correlation, and pre-pregnancy androgen/SHBG ratios showed a positive correlation with birth weight. The correlation between SHBG and birth weight was the strongest and most significant, as compared to the correlation of the T/SHBG, AD/SHBG, and DHEAS/SHBG ratios with birth weight. Pre-pregnancy SHBG level was shown earlier to be a predictor of infant birth weight independent of gestational diabetes and pre-pregnancy BMI [58]. But as differences in birth weight could possibly be ascribed to either real growth potential difference or modified time of birth (since near term, 200–400 g/week growth can still occur), we calculated birth weight percentiles, too. The same parameters remained significantly correlated with birth weight percentile, but the T/SHBG (i.e., FAI) turned out to be more strongly and more significantly correlated with birth weight percentile than SHBG on its own. Thus, the T/SHBG ratio may be slightly more useful as a summed indicator of the growth potential of the fetus than SHBG alone. In our population, pre-pregnancy BMI was not significantly correlated with either birth weight or birth weight percentile (Figure 2).

This study has several strengths. Most importantly, detailed serum androgen levels were analyzed instead of just considering the umbrella diagnosis of PCOS or the clinical symptom of hyperandrogenism. We consider this detailed approach essential in a disease like PCOS, which has many different manifestations and several factors leading to similar symptoms (such as ovulatory disorder or hyperandrogenism). Another advantage of this study is that androgen/SHBG and T/AD ratios were also examined. Thus, we analyzed parameters that potentially provide information about the interaction of the two components used in the ratio or about an actor that affects both (e.g., insulin resistance) or even interconverts them (e.g., the 17-β-HSD enzyme). The single-center character of this study is also an advantage in that diagnosis, treatment, or intervention principles were certainly uniform. Finally, many of the articles cited in this paper were based on infertility treatments of PCOS patients or infertility clinics, whereas our patients were an unselected population of PCOS patients treated for infertility and/or other symptoms of PCOS not only in an infertility clinic but also a reproductive endocrinology clinic, thus allowing broader conclusions and representing better the real-life PCOS population, often seeking medical help outside the scope of infertility treatments.

Some limitations of this study have to be mentioned as well. The retrospective character limited the availability of data. Despite the originally large number of PCOS patients, much less could be included in this study, as several patients did not have the necessary baseline parameters assessed. The reason for this is that androgen levels were often not tested if the diagnosis could be made without laboratory androgen testing (either because ovulatory disorder + PCO morphology on ultrasonography were present or because clinical hyperandrogenism made detailed laboratory testing for androgens unnecessary) or if the exact androgen levels did not have a treatment-modifying significance (e.g., oral contraceptive initiation was planned in mild hirsutism). Some referred patients might have had laboratory tests made elsewhere, and detailed results were not available or mentioned in the documentation. This finally resulted in having small numbers in some subgroups.

In summary, we believe this is the first study to compare detailed pre-pregnancy androgen and SHBG levels, androgen/SHBG ratios, and the 17-β-HSD activity indicator testosterone/androstenedione ratio in various subgroups of obstetric outcome measures following successful pregnancies of PCOS patients. Our results suggest that significant differences exist pre-conceptionally in one or more of the baseline direct or derived androgen parameters between groups having different obstetric complications. It is especially worthy of note that androstenedione and the T/AD ratio seem to be the only difference in some outcomes (miscarriage in the history, preterm delivery, gender of the fetus/newborn), which may suggest some specific role of the altered activity of the 17-β-HSD enzyme, possibly even manifesting in the offspring, too. It is also noteworthy that the pre-pregnancy T/SHBG ratio may be more strongly correlated with the growth potential of the fetus than SHBG itself. These results may help to select PCOS patients requiring enhanced surveillance during their pregnancies and may even determine the direction of this special obstetric attention. On the other hand, these results need to be affirmed in prospective, larger-scale, and well-powered studies in the future.

## Figures and Tables

**Table 1:** Pre-conception androgen levels and occurrence of miscarriage, IVF, gestational diabetes, and preeclampsia.

	Miscarriage in Medical History			Mode of Conception			Gestational Diabetes			Preeclampsia		
	Yes	No	*p*	IVF	Non-IVF	*p*	Yes	No	*p*	Yes	No	*p*
	(Mean ± SD)	(Mean ± SD)		(Mean ± SD)	(Mean ± SD)		(Mean ± SD)	(Mean ± SD)		(Mean ± SD)	(Mean ± SD)	
	n = 14	n = 43		n = 12	n = 48		n = 7	n = 54		n = 9	n = 49	
Testosterone (nmol/L)	1.47 ± 0.47	1.64 ± 0.98	0.56	1.44 ± 1.31	1.59 ± 0.75	0.60	1.84 ± 0.59	1.5 ± 0.90	0.37	1.86 ± 0.99	1.50 ± 0.88	0.28
Androstenedione (ug/L)	**3.60 ± 1.97**	**2.68 ± 1.22**	**0.04**	2.31 ± 1.09	2.93 ± 1.41	0.16	2.81 ± 1.54	2.77 ± 1.36	0.95	3.29 ± 1.47	2.73 ± 1.37	0.29
DHEAS (µmol/L)	8.40 ± 3.71	7.85 ± 3.29	0.60	6.45 ± 3.01	8.05 ± 3.30	0.13	6.77 ± 2.21	7.81 ± 3.38	0.43	9.19 ± 3.32	7.48 ± 3.22	0.17
SHBG (nmol/L)	69.40 ± 54.20	57.77 ± 41.35	0.42	72.01 ± 54.40	54.73 ± 37.85	0.20	38.51 ± 22.58	61.86 ± 44.43	0.18	44.47 ± 21.68	63.08 ± 46.41	0.25
Testosterone/SHBG	0.05 ± 0.09	0.05 ± 0.05	0.80	0.06 ± 0.10	0.04 ± 0.0.4	0.49	0.05 ± 0.05	0.04 ± 0.06	0.66	0.06 ± 0.04	0.04 ± 0.06	0.57
Androstenedione/SHBG	0.07 ± 0.07	0.09 ± 0.11	0.55	0.67 ± 0.08	0.09 ± 0.10	0.48	0.12 ± 0.15	0.08 ± 0.09	0.33	0.11 ± 0.13	0.08 ± 0.09	0.45
DHEAS/SHBG	0.22 ± 0.25	0.22 ± 0.24	0.99	0.20 ± 0.28	0.23 ± 0.23	0.66	0.27 ± 0.26	0.21 ± 0.23	0.56	0.25 ± 0.23	0.21 ± 0.24	0.61
T/AD	0.57 ± 0.26	0.56 ± 0.32	0.93	0.58 ± 0.28	0.59 ± 0.38	0.96	0.66 ± 0.66	0.58 ± 0.31	0.58	0.65 ± 0.22	0.58 ± 0.39	0.64
TSH	2.11 ± 0.83	2.19 ± 1.37	0.81	2.05 ± 0.75	2.00 ± 0.99	0.86	2.50 ± 0.92	1.96 ± 0.92	0.18	2.39 ± 0.74	1.98 ± 0.98	0.26
Age (year)	29.44 ± 3.70	29.18 ± 5.09	0.84	**32.00 ± 5.26**	**28.83 ± 4.44**	**0.027**	**32.22 ± 4.06**	**28.85 ± 4.70**	**0.044**	28.00 ± 6.22	29.69 ± 4.52	0.32
BMI (kg/m^2^)	28.59 ± 6.46	28.11 ± 4.65	0.82	30.17 ± 7.35	28.03 ± 4.79	0.48	28.68 ± 4.09	28.13 ± 5.10	0.82	**32.12 ± 3.05**	**27.66 ± 4.58**	**0.046**

**Table 2:** Pre-pregnancy androgen levels and newborn gender, preterm delivery, and mode of delivery.

	Gender of Newborn			Maturity			Mode of Delivery		
	Female	Male	*p*	Preterm Delivery	Term Delivery	*p*	Vaginal	Cesarean	*p*
	(Mean ± SD)	(Mean ± SD)		(Mean ± SD)	(Mean ± SD)		(Mean ± SD)	(Mean ± SD)	
	n = 31	n = 37		n = 9	n = 52		n = 26	n = 34	
Testosterone (nmol/L)	1.56 ± 0.67	1.57 ± 1.04	0.97	1.57 ± 1.37	1.55 ± 0.81	0.95	1.69 ± 1.00	1.50 ± 0.80	0.41
Androstenedione (ug/L)	**3.57 ± 1.96**	**2.58 ± 1.12**	**0.01**	2.37 ± 1.04	2.91 ± 1.44	0.31	2.85 ± 1.48	2.81 ± 1.31	0.91
DHEAS (µmol/L)	7.50 ± 2.96	8.08 ± 3.59	0.51	7.34 ± 3.08	7.88 ± 3.40	0.67	7.86 ± 3.37	7.78 ± 3.30	0.93
SHBG (nmol/L)	55.84 ± 40.28	62.41 ± 46.84	0.57	77.17 ± 56.51	57.11 ± 41.53	0.21	50.08 ± 30.84	63.89 ± 48.78	0.21
Testosterone/SHBG	0.05 ± 0.04	0.05 ± 0.07	0.97	0.06 ± 0.11	0.04 ± 0.05	0.41	0.06 ± 0.08	0.04 ± 0.04	0.29
Androstenedione/SHBG	0.11 ± 0.12	0.07 ± 0.07	0.16	0.06 ± 0.08	0.09 ± 0.10	0.43	0.10 ± 0.12	0.07 ± 0.07	0.19
DHEAS/SHBG	0.23 ± 0.21	0.22 ± 0.26	0.81	0.22 ± 0.32	0.22 ± 0.23	0.91	0.27 ± 0.26	0.20 ± 0.22	0.29
T/AD	0.50 ± 0.21	0.66 ± 0.45	0.09	**0.82 ± 0.61**	**0.55 ± 0.31**	**0.052**	0.60 ± 0.39	0.58 ± 0.35	0.81
TSH	1.97 ± 0.79	2.09 ± 1.05	0.63	2.15 ± 0.85	1.98 ± 0.95	0.61	**1.77 ± 0.67**	**2.21 ± 1.08**	**0.09**
Age (year)	28.77 ± 3.75	30.05 ± 5.34	0.26	31.20 ± 5.96	29.15 ± 4.50	0.21	28.52 ± 3.98	30.34 ± 5.19	0.10
BMI (kg/m^2^)	28.02 ± 3.92	28.97 ± 4.54	0.55	26.36 ± 6.88	28.62 ± 4.43	0.28	28.53 ± 4.37	28.77 ± 4.33	0.87

**Table 3:** Correlations of pre-pregnancy androgen levels with maternal age at delivery, gestational age, birth weight, and birth weight percentile.

	Maternal Age at Delivery		Gestational Age		Birth Weight		Birth Weight Percentile	
	n = 63		n = 61		n = 61		n = 61	
	r	*p*	r	*p*	r	*p*	r	*p*
Testosterone (nmol/L)	−0.14	0.28	0.11	0.42	0.19	0.15	0.18	0.18
Androstenedione (ug/L)	−0.19	0.16	0.11	0.40	0.18	0.18	0.18	0.18
DHEAS (µmol/L)	**−0.40**	**0.001**	0.15	0.25	0.13	0.32	0.15	0.28
SHBG (nmol/L)	0.13	0.30	−0.19	0.14	**−0.30**	**0.02**	**−0.27**	**0.04**
Testosterone/SHBG	−0.09	0.48	0.07	0.59	**0.28**	**0.03**	**0.28**	**0.03**
Androstendione/SHBG	−0.22	0.08	0.11	0.39	**0.24**	**0.06**	**0.25**	**0.05**
DHEAS/SHBG	**−0.32**	**0.01**	0.15	0.27	**0.26**	**0.04**	**0.27**	**0.03**
T/AD	0.03	0.84	−0.06	0.68	−0.12	0.37	0.11	0.43
TSH	0.06	0.66	0.02	0.89	0.07	0.60	0.03	0.85

## Data Availability

The data presented in this study are available by contacting the corresponding author.
